# Treg Therapies Revisited: Tolerance Beyond Deletion

**DOI:** 10.3389/fimmu.2020.622810

**Published:** 2021-01-28

**Authors:** Nina Pilat, Jonathan Sprent

**Affiliations:** ^1^ Section of Transplantation Immunology, Department of General Surgery, Medical University of Vienna, Vienna, Austria; ^2^ Immunology Division, Garvan Institute of Medical Research, Darlinghurst, NSW, Australia; ^3^ St Vincent’s Clinical School, University of New South Wales, Sydney, NSW, Australia

**Keywords:** regulatory T cells, tolerance, IL-2 complexes, cell therapy, immunotherapy, autoimmunity, transplantation

## Abstract

Induction of immune tolerance is the Holy Grail in transplantation medicine and autoimmunity. Currently, patients are required to use immunosuppressive drugs for the rest of their lives, resulting in unwanted side effects and complication from global suppression of the immune response. It is well established that regulatory T cells (Tregs) are critical for the maintenance of immune tolerance towards self-antigens by several mechanisms of immune regulation, in parallel with intrathymic deletion of self-reactive T cells during ontogeny. Therefore, approaches for increasing Treg numbers or function *in vivo* could provide an all-purpose solution for tolerance induction. Currently, most state-of-the-art therapeutics for treating autoimmune diseases or preventing allograft rejection work either by general immunosuppression or blocking inflammatory reactions and are non-specific. Hence, these approaches cannot provide satisfactory long-term results, let alone a cure. However, in animal models the therapeutic potential of Treg expansion for inducing effective tolerance has now been demonstrated in various models of autoimmunity and allogeneic transplantation. Here, we focus on therapies for increasing the size of the Treg pool by expanding endogenous Treg numbers *in vivo* or by adoptive transfer of Tregs. In particular, we discuss IL-2 based approaches (low dose IL-2, IL-2 complexes) for inducing Treg expansion *in vivo* as well as cell-based approaches (polyclonal, antigen specific, or cell engineered) for adoptive Treg therapy. We also mention new questions arising from the first clinical studies on Treg therapy in the fields of transplantation and autoimmunity.

## Introduction

Foxp3^+^ CD4^+^ regulatory T cells (Tregs) are the key players in the maintenance of peripheral self-tolerance (1, 2). Here, the discovery of FoxP3 as a master regulator for Treg development and function was critical for characterization of these cells and in-depth analysis of Treg biology. A key finding was that mutations in the *FoxP3* gene lead to the development of dysfunctional Treg cells, resulting in severe autoimmunity with early onset of uncontrolled lymphoproliferation in both mice (scurfy mutant) and man (IPEX syndrome). Furthermore, Treg cells have since been demonstrated to be impaired in a variety of autoimmune settings as manifested by a reduction in Treg cell numbers, function, or survival (3).

**Figure 1:**
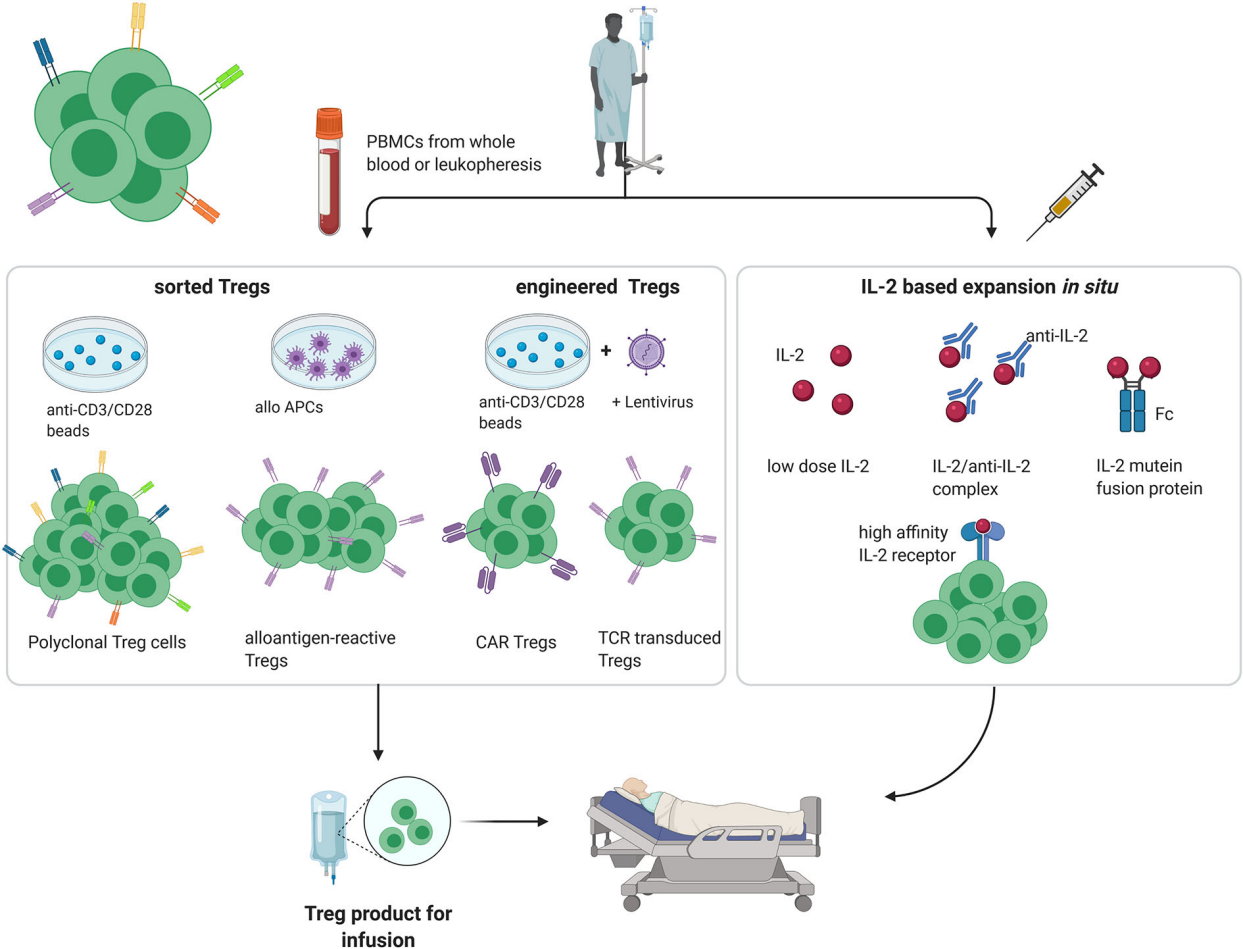
Different approaches for Treg therapy in transplantation and autoimmunity. (PBMC, peripheral blood mononuclear cell; APC, antigen-presenting cell; created with BioRender.com).

In healthy individuals, most immature self-reactive T cells are purged during their development in the thymus *via* negative selection (4). However, especially for tissue-specific antigens, not all self-antigens are displayed in the thymus, with the result that small numbers of self-reactive T cell clones escape into the periphery. Under normal conditions, autoreactivity of these tissue-specific T cells is suppressed by the activity of Tregs. In addition, by multiple mechanisms including synthesis of inhibitory cytokines and reducing the expression of costimulatory molecules on dendritic cells, Tregs play an important role in limiting the intensity of all immune responses, both to self and foreign antigens, thereby preventing immunopathology (5–8). For this reason, amplifying the suppressive function of Tregs is an attractive method for inducing transplantation tolerance. Here, both for autoimmune disease and organ transplantation, several approaches have been described for increasing the Treg : Teff cell ratio to favor tolerance, both in preclinical models and clinical trials (**Figure 1**).

## Adoptive Treg Therapy

The simplest method for increasing the Treg/Teff ratio *in vivo* is to infuse purified populations of Tregs (9). Indeed, pre-clinical studies as well as initial clinical trials have shown that adoptive Treg therapy is a promising therapeutic tool in the treatment of autoimmune diseases and in the induction of tolerance in the field of organ transplantation. However, for routine clinical application of adoptive Treg therapy, two key questions arise:

What is the best source of Tregs for infusion? In most clinical studies, Tregs prepared from peripheral blood mononuclear cells (PBMCs) are used as the starting population for *ex vivo* Treg cell expansion; for prevention of graft-versus-host disease (GvHD) after haematopoietic stem cell engraftment (HSCT), Tregs have also been prepared from umbilical cord blood (10). For HSCT, donor-derived Tregs are most effective in preventing GvHD, whereas for autoimmunity and organ transplantation, recipient-derived Tregs seem to be superior (11).

Do transferred Tregs need to be antigen specific? Although Tregs have to be activated to express their suppressor function, once activated their function is largely non-specific (12). For treatment of autoimmune disease, preclinical data suggest that antigen specific Tregs are superior to polyclonal Tregs in terms of their efficacy and lower risk of pan-immunosuppression (13, 14). However, in preclinical models of autoimmunity, the target antigens vary in their potency to prevent an unwanted immune response (15). Moreover, in human autoimmune diseases, Tregs seem to lack auto-antigen specificity and suppressive phenotype is limited (16). For transplantation tolerance, suppression by polyclonal Tregs can be effective, although Tregs with indirect specificity toward alloantigens have been shown to be preferable (17).

Several reports suggest, that the superiority of antigen specific Tregs is due to specific homing and activation in lymph nodes (15). However, polyclonal Tregs have been shown to prevent effector T cell homing to the graft by modulation of effector T cell trafficking (18). On this point, recent studies suggest that tailoring Treg homing efficiency might be the key to superior suppressor function (19).

The current methods for preparing polyclonal and antigen specific Tregs are considered below.

### Polyclonally-Expanded Tregs

The potency of polyclonal Tregs for tolerance induction by adoptive cell therapy was shown many years ago by Sakaguchi and co-workers who showed that injections of sorted CD4^+^CD25^+^ T cells could rescue mice from organ-specific autoimmune diseases and GvHD-like wasting disease (20). These findings paved the way for comparable studies on Treg therapy for pre-clinical models of GvHD, autoimmune diseases and organ transplantation. For human studies, the first clinical trial of adoptive Treg therapy used purified fresh Tregs taken directly *ex vivo* for the treatment of GvHD after allogeneic HSCT (21). Thereafter, however, most clinical trials have used *ex-vivo* expanded Tregs; such expansion increases not only Treg cell numbers, but also their potency (22). In our hands, *in vitro* expanded polyclonal Tregs are superior to freshly isolated Tregs for induction of chimerism and tolerance in a murine mixed chimerism model (unpublished data of NP).

Polyclonal expansion of Tregs is generally induced by culturing purified CD4^+^ CD25^+^ cells with cross-linked anti-CD3/CD28 antibodies and IL-2 *in vitro* for 1–6 weeks, with most protocols using rapamycin to prevent Teff proliferation [The ONE study (23, 24)]. For efficient suppression of autoimmune disease and allograft survival, the expanded polyclonal Tregs must be injected in large numbers. Here, the main side effect is that increasing the total size of the Treg pool can lead to generalized immunosuppression (25). However, this effect is generally quite minor and substantial “off-target” immunosuppressive effects have not been reported so far.

### Antigen-Specific Tregs

Because of the risk of non-specific immunosuppression, there is increasing interest in the use of antigen-specific Tregs for tolerance induction, especially for solid organ transplantation. Hence, the transplant community is currently placing a lot of effort in the development of efficient approaches for expansion of alloantigen-reactive Tregs (26–29). Various cell culture protocols differing in the type and concentration of stimulator cells (donor derived PBMCs, CD40L activated donor B cells, B cell lines or DCs), the growth factors used and the duration of culture are under intensive investigation. Comparing these methods is difficult, however, because quantitating antigen-specific Tregs is still imprecise and to date has been shown only for freshly isolated Tregs (30).

Importantly, antigen-specific Tregs have been shown to play a major role in HLA-mediated susceptibility and protection of autoimmune diseases (31).

### Engineered Treg Cells: TCR-Transduced Tregs, CAR Tregs

Despite the therapeutic potential of selectively expanding antigen-specific Tregs, cell culture protocols are complex, and the low frequency of the precursor cells tend to limit enthusiasm for this approach. Hence, there is parallel interest in developing methods for engineering Tregs to express antigen-specific TCRs or chimeric antigen receptors (CARs) (32).

TCR-transduced Tregs can be rapidly generated from polyclonal Tregs in large numbers. This is clearly an advantage over expanding Tregs from rare antigen-specific precursor cells, which can take up to 6 weeks of tissue culture. Moreover, TCR-transduced Tregs have been shown to migrate to the target tissue where they exert both antigen-specific and non-specific suppressive effects (33). As mentioned earlier, indirect bystander suppression by Tregs can be highly efficient at promoting allograft survival (17).

With their capacity to recognize native antigen while retaining typical T cytolytic activity, CAR T cells are an invaluable tool for treating hematologic malignancies (34). With further engineering, CAR T cells have been modified to suppress rather than kill target tissues by generating (non-MHC restricted) antigen-specific CAR Tregs from sorted natural Tregs (32, 35). Such CAR Tregs have been shown to prevent xenogeneic GvHD in a humanized murine HSCT model (35). The efficacy and suppressive stability of CAR Tregs has been demonstrated in colitis and skin transplantation models (36); several mechanisms of CAR Treg-mediated suppression have been suggested.

It should be noted that, for both TCR-transduced and CAR Tregs, their production requires retroviral transduction techniques. Hence, their safety for therapy is receiving scrutiny.

## Treg Expansion *In Vivo*

As an alternative to adoptive Treg therapy, Tregs can be selectively expanded *in vivo* by various methods. This approach increases the Treg : Teff cell ratio and allows polyclonally-expanded Tregs to mediate non-specific immunosuppression. This method is simpler and less expensive than adoptive Treg therapy. For organ transplantation, both methods are generally limited to situations where the organ concerned is taken from a living donor.

Several strategies for the *in vivo* induction of Tregs have been described and date back to initial studies on the capacity of anti-CD3 antibody to prolong allograft survival. Thus, treatment with anti-CD3 antibody is now known to induce Treg cell expansion while selectively depleting T effector cells, both in pre-clinical murine (37) and clinical studies (38, 39).

Another approach for Treg induction is to inhibit mTOR function *in vivo*. Thus, it has been shown that injection of rapamycin, an mTOR antagonist, selectively increases endogenous Treg frequency, in parallel with promoting TCR-induced anergy of conventional T cells (40, 41). Moreover, rapamycin has been shown to stabilize the suppressor function and gene expression profile of Tregs, both for endogenous and adoptively-transferred Tregs (42). Although failing as monotherapy, rapamycin was shown to promote long-term persistence of adoptively-transferred Tregs in combination with therapeutic IL-2 in a non-human primate model (43).

As for conventional T cells, Treg expansion and survival depend critically on contact with IL-2 (44). Moreover, manipulating how IL-2 is presented under *in vivo* conditions can be used to either reduce or augment the Treg : Teff ratio, and thereby either augment immunity or induce tolerance. For the latter, below we discuss several approaches that utilize IL-2 for Treg induction *in vivo*.

### Low-Dose IL-2

IL-2 therapy was originally developed for cancer immunotherapy because of its potency to enhance the growth of CD8^+^ T cell and NK cells (45). Despite conspicuous success in some patients, however, therapy with unmodified IL-2 has fallen into disfavor because of severe problems with toxicity. When given in low doses, IL-2 is much less toxic but loses its capacity to stimulate typical cytotoxic cells. Nevertheless, low-dose IL-2 does retain the capacity to stimulate Tregs, reflecting the fact that these cells, unlike CD8 T cells and NK cells, express CD25, the α-chain of the IL-2R. Thus, constitutive expression of the high-affinity IL-2Rαβγ makes Tregs more sensitive to IL-2 than conventional T cells, most of which express low-affinity IL-2Rβγ. For this reason, low-dose IL-2 therapy has emerged as a convenient method for inducing selective expansion of Tregs *in vivo* (46). Tolerance induction by low-dose IL-2 therapy is used mainly for treatment of autoimmunity (47) but is also showing promise for organ transplantation (48) and treatment of GvHD (49).

The major limitation of low-dose IL-2 therapy is that being small, IL-2 is rapidly excreted in the urine and so has a relatively short half-life (<30 min). As discussed below, this problem can be avoided by using IL-2/antibody complexes or IL-2 fusion proteins.

Another problem with IL-2 therapy is that CD25 expression is not unique to Tregs and is also expressed at a lower level on activated T cells. Hence, even in low doses, IL-2 based therapies may cause some level of effector T cell stimulation in addition to Treg expansion.

### IL-2/Anti-IL-2 Complexes

Studies on the effects of anti-IL-2 monoclonal antibodies (mabs) *in vivo* showed that injecting mice with IL-2 bound to particular IL-2 mabs, notably JES6-1, led to selective expansion of Tregs and the onset of immunosuppression (50). The capacity of IL-2/mab complexes to enhance allograft survival was demonstrated in a murine model of islet transplantation (51). Thus, short-term IL-2/mab complex treatment led to indefinite survival of >80% of islet allografts without any immunosuppression. When combined with rapamycin, these complexes were also potent at preventing induction of experimental autoimmune encephalomyelitis (EAE) and other autoimmune diseases (51, 52).

Despite prolonging the survival of islet allografts, IL-2 mab complexes failed to augment the survival of skin allografts as monotherapy (53). However, later experiments showed that modification of the treatment protocol led to prolonged skin allograft survival. Thus, when IL-2/mab complexes were supplemented with rapamycin and injected for 30 days, accompanied initially with short-term anti-inflammatory treatment with anti-IL-6 mab, survival of fully-mismatched murine skin allografts was improved from 15 days to 85 days (53). Treg levels returned to near-normal levels in the lymphoid tissues soon after the injections were stopped; however, intragraft Treg levels remained elevated for several weeks (53). Notably, although the grafts were eventually rejected, donor antibody formation was minimal. This and other findings suggested that prolonged graft survival was largely a reflection of Treg-meditated immunological ignorance. With regard to clinical relevance, the efficacy of IL-2/mab therapy is currently being investigated in a murine model of cardiac transplantation; in preliminary experiments, the results have shown long-term allograft survival and prevention of chronic allograft vasculopathy (unpublished data of NP).

### IL-2 Fusion Proteins and Muteins

As with pegylation and binding to albumin, fusing IL-2 with the Fc portion of IgG (IL-2-Fc) retards excretion and thereby considerably increases the half-life of IL-2. For low-dose IL-2 therapy, IL-2 fused to non-FcRγ binding human IgG1 has been shown to be superior to unmodified IL-2 for the induction of Tregs in a non-human primate model (54).

Based on the binding interaction of IL-2 with IL-2 mabs, IL-2 can be mutated to selectively stimulate either CD8 T cells and NK cells or Tregs (45). For Treg stimulation, IL-2 is mutated to bind poorly to IL-2R γ or β chains with retention of normal or above-normal binding to the α chain (55). These IL-2 muteins thus resemble IL-2/JES6-1 mab complexes in preferentially stimulating Tregs. When prepared as IL-2-Fc fusion proteins to increase half-life, these IL-2 muteins were superior to wild-type IL-2-Fc in the treatment of type I diabetes (T1D) in a pre-clinical mouse model (56).

## Clinical Studies

It is now more than a decade since the first clinical trial using adoptive Treg therapy for the treatment of GvHD after allogeneic HSCT (21), which was followed by several early-phase trials focusing on safety, feasibility and tolerability of Treg infusions. Most of these phase I or phase I/II trials used polyclonally expanded Tregs from PBMCs and have been conducted in the setting of GvHD, new-onset T1D and solid organ transplantation (36).

Results of published and ongoing clinical trials in the setting of autoimmunity are summarized elsewhere (57). Notably, the first published trials using Treg therapy for T1D have reported cessation of disease progression or even remission of disease in some patients, promising not only safety and feasibility but also efficacy. Ongoing clinical trials suggest that effective tolerance protocols *via* Treg therapy will soon be available for several types of autoimmune disease. Data on long-term results are still very limited, however, and many questions remain open, including which cell product/expansion strategy is optimal in terms of cell yield/specificity/potency, and the number and frequency of Treg cell injections required for efficient tolerance induction. Hopefully, the ongoing clinical trials will resolve these questions.

For solid organ transplantation, a large number of clinical trials using Treg therapy for tolerance induction are ongoing. These trials are summarized in **Table 1** and involve both polyclonal and antigen-specific Treg infusion.

**Table 1:** Ongoing clinical trials adopting Tregs for tolerance induction in transplantation (search date Oct 1^st^ 2020, listed in clinicaltrials.gov; LUP, last update posted).

Study ID	Phase	Ages eligible	Product	Dose	Status (LUP)	Actual enrollment (estimated)	Title	Location
*Renal Transplant—adoptive cell therapy*								
NCT02088931	I	18-50 (adult)	Autologous polyclonally expanded CD4+CD127lo/-CD25+ Tregs	3.2 x 10^8^	Active, not recruiting (Oct 2016)	3	Treg Adoptive Therapy for Subclinical Inflammation in Kidney Transplantation (TASK)	University of California, San Francisco, US
NCT02091232	I	>18 (adult, older adult)	Recipient Tregs stimulated with donor PBMCs and belatacept	4–9 x 10^8^	Completed (Aug 2020)	5 (8)	Infusion of T-Regulatory Cells in Kidney Transplant Recipients (The ONE Study)	Massachusetts General Hospital, Boston, US
NCT03943238	I		Autologous polyclonally expanded Tregs	25 x 10^6^ starting dose (dose escalation)	Recruiting (March 2020)	n/a (22)	TLI, ATG & Hematopoietic Stem Cell Transplantation and Recipient T Regs Therapy in Living Donor Kidney Transplantation	Stanford University, Palo Alto; Northwestern University, Chicago, US
NCT03284242	n/a	18-65 (adult, older adult)	Autologous polyclonally expanded Tregs	n/a	Recruiting (Feb 2020)	n/a (12)	A Pilot Study Using Autologous Regulatory T Cell Infusion Zortress (Everolimus) in Renal Transplant Recipients	University of Kentucky, Lexington, US
NCT02711826	I/II	>18 (adult, older adult)	Autologous polyclonal Tregs	1 x 10^6^—1 x 10^7^	Recruiting (May 2020)	n/a (30)	Treg Therapy in Subclinical Inflammation in Kidney Transplantation (TASK)	University of California, San Francisco, US And others (8 centers)
NCT02145325	I	18-65 (adult, older adult)	Autologous polyclonally expanded CD4+CD25+ nTregs	0.5–5 x 10^9^	Completed (Oct 2019)	10 (12)	Trial of adoptive Immunotherapy with TRACT to prevent rejection in living donor kidney transplant patients	Northwestern University Comprehensive Transplant Center, Chicago, US
NCT03867617	I/II	>18 (adult, older adult)	Autologous polyclonally expanded CD4+CD127lo/-CD25+CD45RA Tregs	0.3–1.5 x 10^7^	Recruiting (Sept 2020)	(12)	Cell Therapy for Immunomodulation in Kidney Transplantation	Medical University of Vienna, Vienna, Austria
NCT 01446484	I/II	1-18 (child)	Autologous polyclonally expanded CD4+CD25+CD127lowFoxP3+ Tregs	2 x 10^8^	Unknown (Nov 2011)	(30)	Treatment of Children With Kidney Transplants by Injection of CD4+CD25+FoxP3+ T Cells to Prevent Organ Rejection	Russian state Medical University, Moscow, Russian Fed
NCT 02371434	I/II	18-65 (adult, older adult)	autologous polyclonally expanded CD4+CD25+FoxP3+ nTregs	0.5–3 x 10^6^	Completed (Feb 2020)	17	The ONE Study nTreg Trial (ONEnTreg13)	Charite University Medicine, Berlin, Germany
NCT02244801	I	18-70 (adult, older adult)	Donor alloantigen reactive Tregs (darTregs)	3 x 10^8^; 9 x 10^8^	Completed (Oct 2018)	6	Donor-Alloantigen-Reactive Regulatory T Cell (darTreg) Therapy in Renal Transplantation (The ONE Study)	University of California, San Francisco, US
NCT02129881	I/II	18+ (adult, older adult)	Autologous polyclonally expanded Tregs	1–10 x 10^6^/kg	Completed (Jan 2019)	15	The ONE Study UK Treg Trial (ONETreg1)	Kings College Hospital London, United Kingdom
*Renal Transplant—endogenous Treg expansion*								
NCT02417870	I/II	18-75 (adult, older adult)	Low-dose recombinant IL-2 (proleukin)		Unknown (Jul 2017)	(5)	Ultra-low Dose Subcutaneous IL-2 in Renal Transplantation	Brigham and Women´s Hospital, New Boston, US
*Liver Transplant—adoptive cell therapy*								
NCT01624077	I	10-60 (child, adult)	autologous polyclonally TGFb induced CD4+CD25+CD127- Tregs	1 x 10^6^/kg	Unknown (Feb 2015)	1	Safety Study of Using Regulatory T Cells Induce Liver Transplantation Tolerance	Nanjing Medical University Nanjing, china
NCT03654040	I/II	18-70 (adult, older adult)	Autologous expanded alloantigen reactive Tregs (arTregs)	0.3–5 x 10^8^	Not yet recruiting (Jul 2020)	(9)	Liver Transplantation With Tregs at UCSF (LITTMUS-UCFS)	University of California, San Francisco, US
NCT03577431	I/II	18-70 (adult, older adult)	Autologous expanded donor alloantigen specific CD4+CD25+CD127- Tregs (arTregs)	(1) 2.5–500 x 10^6^	Recruiting (Oct 2019)	(9)	Liver Transplantation With Tregs at MGH (LITTMUS-MGH)	Massachusetts General Hospital, Boston, US
NCT02474199	I/II	18-70 (adult, older adult)	Autologous donor alloantigen reactive Tregs (darTregs)	3–5 x 10^8^	Completed (Jan 2020)	14 (18)	Donor Alloantigen Reactive Tregs (darTregs) for Calcineurin Inhibitor (CNI) Reduction (ARTEMIS)	University of California, San Francisco, US Northwestern University Comprehensive Transplant Center, Chicago, US Mayo Clinic in Rochester Rochester, US
NCT02188719	I	21-70 (adult, older adult)	Autologous donor alloantigen reactive Tregs (darTregs)	2.5–6 x 10^7^	Terminated (Aug 2020) – has results	15	Donor-Alloantigen-Reactive Regulatory T Cell (darTregs) in Liver Transplantation (deLTa)	University of California, San Francisco, US Northwestern University Comprehensive Transplant Center, Chicago, US Mayo Clinic in Rochester Rochester, US
NCT02166177	I/II	18-70 (adult, older adult)	Autologous polyclonally expanded Tregs	0.5–1; 3–4.5 × 10^6^/kg	Completed (Jan 2019)	9	Safety and Efficacy Study of Regulatory T Cell Therapy in Liver Transplant Patients (ThRIL)	Kings College Hospital London, United Kingdom
*Liver Transplant—endogenous Treg expansion*								
NCT02739412	II	18-65 (adult, older adult)	Low-dose recombinant IL-2 (proleukin)	0.30 MIU per meter squared body surface area for 4 weeks	Active, not recruiting (Jan 2020)	7	Efficacy of Low Dose, SubQ Interleukin-2 (IL-2) to Expand Endogenous Regulatory T-Cells in Liver Transplant Recipients	Beth Israel Deaconess Medical Center, Boston, US
NCT02949492	IV	18-50 (adult)	Low-dose recombinant IL-2 (proleukin)		Terminated (Aug 2019)	6	Low-dose IL-2 for Treg Expansion and Tolerance (LITE)	Kings College Hospital London, United Kingdom

In these studies, Treg isolation under GMP conditions was carried out with the “CliniMACS” system (CliniMACS TM Instruments, Miltenyi Biotec) which involves clinical-scale magnetic enrichment of cells (CD8 depletion followed by CD25 enrichment) in a closed and sterile system, or, outside European Union countries, by flow sorting of cells for expression of CD4, CD25, and CD127 (yielding populations of >99% purity). For preparation of antigen specific Tregs, magnetic bead isolation may be superior to flow-sorting of Tregs because contamination with residual antibodies impairs Treg expansion (26), perhaps by interfering with Treg : APC contact during culture (58).

The results of these trials will clearly be awaited with great interest. The results are not easy to predict because, in contrast to mice, inducing tolerance in humans is proving to be especially difficult. This issue is discussed below.

## Lessons Learned: The Complexity of Transplantation Tolerance in Humans

For autoimmune diseases, Treg based therapies are promising and have shown initial success for treatment of T1D, SLE and inflammatory bowel disease [(59, 60) and reviewed in (61)]. For solid organ transplantation, however, the results are less clear, perhaps reflecting that the precursor frequency of T cells recognizing allo-MHC is very high (up to 10%) (62, 63). For this reason, employing Treg therapy alone to induce allograft tolerance may be a challenging task. Moreover, as discussed below, there is the additional problem that effective models for tolerance inductions in mice are not necessarily applicable to clinical transplantation. Thus, it has to be borne in mind that typical mouse studies are generally based entirely on a single inbred strain housed in a “clean” environment; also, memory T cells, which are more difficult to regulate than naïve cells, are less common in clean mice than humans (64).

Despite the importance of Tregs, long-term tolerance to all antigens, including alloantigens, requires efficient elimination of reactive T cells in the thymus during ontogeny (65). In pre-clinical models, efficient tolerance to organ allografts generally requires hematopoietic chimerism, which is induced by transfer of donor stem cells after myelosuppressive treatment of the host to remove donor-reactive mature T cells (66). Although the mixed chimerism approach is also successful in a clinical setting (67), the side effects of this procedure are considerable (68). For this reason, there is much interest in developing mixed-chimerism approaches that avoid heavy immunosuppression of the host (69). For the protocol for HLA-disparate renal allografts developed by investigators at Massachusetts General Hospital (MGH), the transplant hosts are conditioned with non-myeloablative immunosuppression followed by combined kidney and HSCT. The results of this approach are promising and have shown long-term graft acceptance in a proportion of patients, with no evidence of GvHD. These findings are surprising because donor cell chimerism is only transient, implying that long-term graft survival involves some form of immunoregulation. Indeed, ongoing studies have shown that persistence of tolerance when chimerism wanes depends crucially on continuous contact with the donor kidney (70), such contact causing progressive elimination of graft-reactive effector T cells in parallel with expansion of graft-specific Tregs (71, 72).

These clinical data are intriguing and are in line with comparable studies in various preclinical models (73, 74). Overall, the results highlight the view that induction and maintenance of tolerance is remarkably complex and involves the combined effects of clonal deletion (affecting both immature and mature T cells) and specific suppression as well as bystander immunoregulation by Tregs. In addition, it is important to bear in mind that, for organ transplantation, tolerance protocols designed for particular organs may not be successful for other transplants. Thus, it was mentioned earlier that polyclonal Treg expansion in mice is much more tolerogenic for islet than skin allografts. Similarly, non-human primate studies have shown that the MGH mixed-chimerism protocol for renal allografts is much less successful for transplantation of other organs (70). There is also the enigma that tolerance induction to allografts is intrinsically difficult for some organs such as skin or intestines but relatively easy for certain other organs, notably the liver (75, 76). Whether this difference is simply related to organ size or has other explanations is still unclear (77).

## Outlook

Despite several decades of research in tolerance induction and Treg therapy in animal models, clinical trials with these models are still uncommon. For autoimmune disease, there is reason for optimism because early trials with Treg therapy are encouraging in terms of both safety and efficacy; moreover, new methods of genetic engineering to prepare antigen-specific Tregs and modify IL-2 to promote their survival *in vivo* show considerable promise (61, 78). However, many questions remain, including the range of autoimmune diseases suitable for Treg therapy. Thus, therapies that work well for one disease, e.g. multiple sclerosis, might work poorly for other diseases, e.g. systemic lupus erythematodes (SLE). In addition, there is the problem that many patients with autoimmune disease are routinely treated with immunosuppressive drugs, making it challenging to evaluate the effect of Treg cell therapy in the presence of these drugs. For these reasons, progress in employing tolerance protocols to treat autoimmune disease is likely to be slow and involve studies on multiple types of disease, both in animal models and clinical trials.

In the field of organ transplantation, using tolerance protocols to prevent rejection is still largely experimental because the current approach of continuous maintenance on calcineurin inhibitors and/or other immunosuppressants is remarkably successful. Nevertheless, the increased incidence of malignancy with this treatment as well as uncontrollable chronic rejection continues to elicit interest in devising methods for long-term tolerance induction without immunosuppression, especially in younger patients. Considering the success of permanent tolerance induction *via* mixed chimerism in animal models, it is clearly disappointing that achieving permanent chimerism without the risk of GvHD in a clinical setting is still not possible. However, the finding that even transient chimerism can be followed by prolonged graft survival and operational tolerance with renal allografts is clearly encouraging. Here, we envisage that, although Treg therapy alone might be insufficient to allow long-term allograft survival, combining Treg therapy with other forms of alloimmune response suppression will significantly boost tolerance induction, especially with antigen-specific Tregs and the use of IL-2 muteins to maintain their survival. Clearly much more research is needed in this area.

## Data Availability Statement

The original contributions presented in the study are included in the article/supplementary material. Further inquiries can be directed to the corresponding author.

## Author Contributions

NP and JS designed the concept and wrote the manuscript. All authors contributed to the article and approved the submitted version.

## Funding

This work was supported by the Austrian Science Fund (P31186-B28 to NP) and grants from the National Health and Medical Research Council of Australia to JS.

## Conflict of Interest

The authors declare that the research was conducted in the absence of any commercial or financial relationships that could be construed as a potential conflict of interest.
